# Complete Genome Sequence of an MLB2 Astrovirus from a Turkish Child with Diarrhea

**DOI:** 10.1128/genomeA.00619-13

**Published:** 2013-08-15

**Authors:** Marcelo Takahiro Mitui, Gulendam Bozdayi, Takashi Matsumoto, Buket Dalgic, Akira Nishizono, Kamruddin Ahmed

**Affiliations:** Department of Microbiology, Faculty of Medicine, Oita University, Yufu, Oita, Japana; Department of Medical Microbiology, Faculty of Medicine, Gazi University, Ankara, Turkeyb; Department of Pediatric Gastroenterology, Faculty of Medicine, Gazi University, Ankara, Turkeyc; Research Promotion Institute, Oita University, Yufu, Oita, Japand

## Abstract

Only two complete genome sequences of MLB2 astroviruses are available, one from an Indian child with diarrhea and another from plasma of an American child. Here we report the complete MLB2 genome sequence from a Turkish child with diarrhea. This MLB2 astrovirus genome sequence shows high nucleotide identity with the American MLB2.

## GENOME ANNOUNCEMENT

The prevalence of astroviruses varies remarkably: 1.9% in Saudi Arabia (1), 3% in Australia (2), 3.4% in Guatemala (3), 4.9% in Spain (4), 8.6% in Thailand (5), 52% in India (6), and 61% in Mexico (7). With eight genotypes, human astroviruses (HAstVs) are highly divergent. Two additional clades have been detected, HAstV-MLB and HAstV-VA, which contain the genotypes MLB1-3 and VA1-VA4 (8). Complete genome sequences of MLB2 are rare; so far only two have been reported, one from an Indian child with diarrhea and the other from the plasma of an American child with an fever of unknown origin (9, 10). Therefore, there is a gap in our understanding of the epidemiology and evolution of this virus. In most countries, no systematic search for astroviruses has been attempted, and no etiologic agent has been found in about 40% of diarrheal cases (9). Turkey is situated in the crossroads of Asia, Europe, and Africa, and thus has the potential to harbor infections of many different origins. Most studies regarding viral gastroenteritis in Turkish children are on rotaviruses (11). Reports of other viruses causing diarrhea in Turkey are scarce, so in this study we aimed to identify astroviruses in children with diarrhea.

Genomic RNA was extracted from 150 rotavirus-negative stool samples collected between September 2004 and December 2005 from children under 5 years of age with diarrhea attending Gazi University Hospital (GUH) and the Ministry of Health, Ankara Training and Education Hospital (11). These samples were subjected to PCR amplification for human astroviruses (12). The primers used for complete genome sequencing of MLB2 were designed using reported sequences (9, 10). The terminal parts were amplified using a rapid amplification of cDNA ends kit, and the PCR products were purified and sequenced (13).

An astrovirus MLB2 was identified by BLAST searching and designated GUP187. This was collected in November 2005 from a 24-month-old girl attending the outpatient department of GUH. The complete GUP187 genome sequence has the highest identity (98%) with MLB2 strain MLB2/human/Stl/WD0559/2008 (GenBank accession no. JF742759) reported from the United States. Excluding the 3′-poly(A) tail, the complete genome sequence of GUP187 comprises 6,119 nucleotides (nt) with three open reading frames (ORFs), ORF1a, ORF1b, and ORF2, with lengths of 2,361 (residues 15 to 2378), 1,533 (2315 to 3850), and 2,235 (3843 to 6080) nt, respectively. The 5′- and 3′-end untranslated sequences were 14 nt and 42 nt, respectively. Compared with strain MLB2/human/Stl/WD0559/2008, the nucleotide (amino acid) identities of ORF1a, ORF1b, and ORF2 of strain GUP187 were 98% (99%), 99% (99%), and 98% (99%), respectively. The 5′- and 3′-end untranslated sequences were identical between strains GUP187 and MLB2/human/Stl/WD0559/2008. When the nucleotide sequence of strain GUP187 was aligned with those of other MLBs, we found a highly conserved core region of 52 nt at the junction between ORF1b and ORF2, showing a 100% identity with MLB2 and MLB3 strains, whereas the identity was 84.6% with MLB1 strains. GUP187 has a heptameric slippery sequence, which is conserved in residues of 2,330 to 2,336 nt (14).

### Nucleotide sequence accession number.

The complete genome sequence of GUP187 appears in the DDBJ/EMBL/GenBank database with the accession number AB829252.
